# Implications of Neural Plasticity in Retinal Prosthesis

**DOI:** 10.1167/iovs.63.11.11

**Published:** 2022-10-17

**Authors:** Daniel Caravaca-Rodriguez, Susana P. Gaytan, Gregg J. Suaning, Alejandro Barriga-Rivera

**Affiliations:** 1 Department of Applied Physics III, Technical School of Engineering, Universidad de Sevilla, Sevilla, Spain; 2 Department of Physiology, Universidad de Sevilla, Sevilla, Spain; 3 School of Biomedical Engineering, University of Sydney, Sydney, Australia

**Keywords:** visual prosthesis, neural plasticity, retinal implant

## Abstract

Retinal degenerative diseases such as retinitis pigmentosa cause a progressive loss of photoreceptors that eventually prevents the affected person from perceiving visual sensations. The absence of a visual input produces a neural rewiring cascade that propagates along the visual system. This remodeling occurs first within the retina. Then, subsequent neuroplastic changes take place at higher visual centers in the brain, produced by either the abnormal neural encoding of the visual inputs delivered by the diseased retina or as the result of an adaptation to visual deprivation. While retinal implants can activate the surviving retinal neurons by delivering electric current, the unselective activation patterns of the different neural populations that exist in the retinal layers differ substantially from those in physiologic vision. Therefore, artificially induced neural patterns are being delivered to a brain that has already undergone important neural reconnections. Whether or not the modulation of this neural rewiring can improve the performance for retinal prostheses remains a critical question whose answer may be the enabler of improved functional artificial vision and more personalized neurorehabilitation strategies.

##  

New technological advances are promising a future cure for blindness. In particular, people diagnosed with retinal degenerative diseases such as retinitis pigmentosa may soon benefit from a repertoire of therapies of different technological bases.^1^ On the one hand, biotechnological approaches are progressing rapidly toward the bedside with clinical studies being carried out in the field of stem cell transplants^2^^–^^5^ and optogenetics,^6^ among others. An example of this recent success is the use of viral vectors to repair specific genetic defects, particularly in the human retinal pigment epithelium-specific 65-kDa (RPE65) gene.^7^ Although similar technologies may now face a faster track for approval after the proliferation of novel vaccines for the pandemic produced by the global transmission of the severe acute respiratory syndrome coronavirus 2 virus,^8^ there are ethical principles that must govern their development^9^ and cannot be understated or circumvented. For instance, despite the promising potential of the clustered regularly interspaced short palindromic repeat/Cas9 technique (CRISPR/Cas9) for curing some types of genetic retinal conditions,^10^^,^^11^ there are also major concerns regarding its safety, particularly because it has the potential to cause secondary mutations.^12^ On the other hand, several visual prostheses have been developed^13^ inspired by the undeniable success of the cochlear implant.^14^ There are four major approaches: (1) retinal implants (subretinal, epiretinal, and suprachoroidal),^15^^,^^16^ (2) stimulation of the optic nerve,^17^^,^^18^ (3) stimulation of the lateral geniculate nucleus (LGN),^19^^,^^20^ and (4) cortical prostheses.^21^^–^^23^ All of these technologies have a common underlying working principle, that is, the activation of the surviving neurons of the visual system to elicit a visual sensation. However, after progression of vision loss, neither the retina nor the visual centers of the brain remain the same, as neural remodeling occurs.^24^^,^^25^ In addition, the visual information sent to the brain differs from that in physiologic vision.

Retinal remodeling takes place as a consequence of the degradation of the photoreceptors.^26^ Among others, this remodeling includes the formation of abnormal neural circuits and the alteration of the electrophysiologic properties of the retinal pathways. The surviving neurons of the retina can then be activated, for example, by electrical stimuli delivered from a retinal prosthesis to restore some degree of visual sensation. The neural patterns thus elicited originate from a substantially different neural circuitry in the degenerated retina.^27^ Furthermore, the neural activation patterns elicited by classical biphasic pulses differ substantially from those in normal vision,^28^ as different neural pathways are typically activated simultaneously in the currently available retinal prostheses. Some studies have shed light on the possibility of providing some degree of selective activation using classical stimulus waveforms.^25^^,^^29^^,^^30^ Other studies suggest the use of high-frequency stimulation to achieve said goal. However, despite the recent progress in preferentially activating the ON and OFF pathways using high-frequency stimulation,^31^^–^^33^ these advances have not yet been demonstrated in vivo.

It is important to note that the remodeling that occurs in the retina does not happen alone. During progression of blindness, the visual cortex (VC) does not go unused. Instead, other sensory areas, typically devoted to processing touch^34^ or hearing,^35^ recruit neurons in the VC to process nonvisual information. These cross-modal neuroplastic changes occur as an adaptive mechanism to sensory deprivation or loss; whether they are beneficial or deleterious in the rehabilitation process of seeing again remains to be investigated.^36^^–^^39^

Here, we aim to provide a descriptive literature review on two phenomena that are limiting the progress in the race for restoring sight, with a clear focus on their implications to retinal prostheses. The neuroplastic transformation that takes place in the visual system determines the starting point from which a retinal prosthesis is deployed to restore vision. In addition, currently available technologies are unable to replicate the neural code of the physiologic vision, and consequently, wrong neural information is being sent to higher visual centers for interpretation. In other words, current devices are sending abnormal neural patterns to an altered brain that is no longer as that of a sighted person.

### Available Animal Models in Bionic Vision Research

Visual perception begins at the retina, a multilayer structure able to generate different neural patterns as a response to the incidence of photons scattered from a visual scene. Each cell layer within the retina has developed a specific function in shaping the neural information: reception, modulation, and transmission of visual information to the brain. Briefly, incident light hyperpolarizes the photoreceptors, producing a reduction in the neurotransmitter release to the dendrites of the bipolar cells causing their subsequent activation. Each photoreceptor connects to several bipolar cells. Individually, these bipolar cells are specialized in coding a specific trait of the whole visual input. The information integrated by bipolar cells gets modulated by the information from surrounding bipolar and amacrine cells and is finally delivered to a spectrum of different types of retinal ganglion cells (RGCs).^40^ Each RGC type will then trigger action potentials (APs) that depend on particular features of the afferent signals. Visual information encoded by the RGCs is eventually delivered to higher visual centers in the brain through the optic nerve for interpretation.^41^ In primates, the most important RGC types are midget and parasol cells. These cells comprise between 60% and 70% of the primate retina^42^ and deliver visual information to the LGN directly, which is subsequently processed in the VC and expressed as a visual perception (see Field and Chichilnisky^43^ for a review).

A wide variety of animal models have been used to unravel the retinal function and to understand the histologic and physiologic changes that occur in the retina and visual processing centers in the brain during blindness. Animal models, traditionally cats,^44^^,^^45^ sheep,^46^^,^^47^ rabbits,^48^ or nonhuman primates,^49^ have been also employed to test retinal prosthesis and electrical stimulation strategies. However, murine models (rats and mice) have gained relevance among research groups in the field, particularly for in vitro research, as they offer a wide variety of strains with various retinal degeneration phenotypes that mimic the diseased retina in the human being. Among others, murine models allow easily replicable in vivo approaches, enabling the possibility to study the electrophysiologic responses arising in the brain following retinal stimulation in dystrophic and nondystrophic experimental groups.^50^

Despite the evident benefits from using murine models, a fundamental question on whether research results are fully translational to humans needs to be taken into account. The most important differences between murine models and primate, both human and nonhuman, are that the former lack an anatomic fovea and saccadic eye movements,^51^ showing a fundamental divergence in the visual strategy between both groups: while murine vision relies on a general interpretation of the visual scene, vision in primates combines this general perception (corresponding to the largest portion of the retina) with a fine and detailed vision provided by the fovea. This involves important differences in the neuronal organization of the retina^52^ and, presumably, in the upstream neural circuitries and processing centers. However, a recent study in mice has shown that despite the lack of histologic evidence of a fovea-like high-resolution structure at retinal level, the VC seems to have an enhanced resolution area corresponding to a specific region of the retina.^53^ Furthermore, these studies have been able to demonstrate that these animals do execute compensatory eye movements in an attempt to maintain this extra-sensitive retinal area aligned to a certain area of interest while they freely move within a given environment.

Also, it is important noting that although the basic neural organization of the retina is highly maintained among mammals,^54^^,^^55^ each animal group possesses unique cell subtypes; more than 32 different RGC types, classified as nondirection-selective and direction-selective ON, OFF, and ON-OFF cells,^56^ and approximately 15 different bipolar cell types have been identified in the mouse retina.^57^ In primates, these numbers are approximately 18 and 12 for RGCs and bipolar cells, respectively.^58^^,^^59^ Thus, despite mice and rats being good candidates for the study of the biological basis underlying the impact of retinal degenerative diseases along the visual pathways (including the visual processing centers in the brain), human visual perception might be strikingly different. For that reason, future research on nonhuman primates, with a more similar visual system to that of humans, might be needed before novel approaches are tested in humans.

## Changes in the Retina Produced by Retinal Degeneration

### Retinal Reorganization

The neural organization of the retina gets endangered as a consequence of the death of photoreceptors, a typical manifestation that occurs during progression of retinal degenerative diseases such as retinitis pigmentosa. The absence of synaptic connections between the photoreceptors and the inner neurons induces a rewiring process that propagates downstream along the retinal layers and leads eventually to the formation of abnormal neural circuits^60^ that can ultimately affect the RGCs.^61^^–^^63^

The retinal degeneration process undergoes three differentiated phases that are mainly characterized by (1) molecular changes that occur within photoreceptors and alterations in bipolar glutamate receptors; (2) death of photoreceptors, bipolar cell deafferentation, and Müller cell hypertrophy; and (3) cell migration to other layers and the formation of microneuromas.^64^ During the second and third degeneration phases, a generalized function switch occurs in all ON-rod bipolar cells lacking synaptic inputs, thus becoming OFF cell types.^64^ At a synaptic level, it has also been observed that these ON rod bipolar cells establish thereafter a faint connection with their nearest cone, as revealed by excitation maps obtained using kainic acid.^60^^,^^65^^,^^66^ Alongside, studies on rd1 mice have also shown connections switches in the dendritic arbors of the RGCs, typically in the connections with bipolar cells within the inner plexiform layer.^67^ This is particularly remarkable because, as ON and OFF bipolar cell axon terminations are layered separately, RGCs could be now receiving the opposite intended input from bipolar cells.

The visual prosthesis research community has recently advanced toward closing the gap between artificial and physiologic neural encoding of the visual information.^68^ Despite the many retinal neuron subtypes currently identified, ON and OFF bipolar and RGCs have been chosen as the main targets to elicit preferential activation, as described later on. The underlying reason is that ON/OFF cells display opposing behaviors: the former activates when the light goes on, whereas the later activates when the light ceases.^69^ Therefore, the question on how retinal degenerative diseases alter the ON and OFF neural population types should be thoughtfully considered, as it may have relevant implications on the electrical stimulation strategies required to produce optimal neural patterns.

### Modification of the Electrophysiologic Properties

The rewired retina entails significant changes in its physiologic properties that affect its responsiveness to visual and electrical stimulation.

Spontaneous activity is an aberrant behavior found in the degenerated retina characterized by increased RGC activity and a low-amplitude and low-frequency oscillatory (∼5 mV, ∼10 Hz) synaptic activity.^70^ Research on rd1 and rd10 mice shows that it arises from an electrically coupled network established between AII amacrine and ON cone bipolar cells.^70^^–^^72^ Most of the biological principles underlying said spontaneous activity have already been reported. The focus is now placed on mitigating the presumably deleterious effects that these spontaneous firing patterns could have in the quality of the regained visual perception (see Trenholm and Awatramani^73^ for a review). In this context, the extent to which spontaneous activity hampers the transmission of light-driven neural responses needs to be clarified (see Stasheff^74^ for a review). Experiments in the rd10 model showed that the emergence of spontaneous hyperactivity does not compromise the ability of the remaining neural pathways with viable photoreceptors to properly respond to light.^75^ However, the blockade of gap junctions, a protein structure that establishes connections between adjacent cells, has been shown to produce a mitigation of this excessive spiking activity; once this background activity has been reduced, an increase in the sensibility of the retina to light and electrical stimuli was reported^76^^,^^77^—that is, a reduction of the activation thresholds. Similarly, a recent study showed that this spontaneous activity can also be abolished by delivering an electrical prestimulus to the retina^78^; this would reduce the activation threshold to a subsequent stimulus. In both approaches, the reduction of the spontaneous activity seems to be the underlying strategy that facilitates improved retinal responses. Further studies need to be carried out in this direction to clarify whether this improved retinal activation relates to more meaningful visual percepts.

Retinal spontaneous firing rate has been shown to change with postnatal age in rd10 individuals,^75^^,^^79^ thus establishing a link between said spontaneous retinal activity and the degenerative stage of the retina. Furthermore, the responses of the RGCs to electrical stimulation have been shown to be influenced by said degeneration stage.^80^ This has evidenced the potential relevance of adapting electrical stimulation strategies to the stage of retinal degeneration. In this direction, Park et al.^81^ proposed a new parameter that measures the sensitivity of the retina to electrical stimulation depending on its degeneration stage. This type of measurement can be used to assess the suitability for receiving a retinal implant.

Note that it is important to distinguish between those stimulation strategies that target the RGCs directly (direct activation) and those that activate primarily the neurons in the retinal network (indirect activation of the RCGs). Note also that indirect activation requires bipolar and amacrine cells, as well as their synaptic connections, to be preserved. Therefore, the retinal rewiring that takes place during retinal degeneration would influence the final neural pattern delivered to the RGCs. However, the integrity of the inner retinal layers does not have a major impact on those stimulation strategies that target RGCs directly, as long as RGCs are sufficiently preserved. As evidence of this, no differences have been reported in the activation thresholds between P23H-1 rat retinae and the wild-type control when activating the RCGs directly.^82^ However, indirect activation experiments showed that higher current levels were required to evoke responses in the rd1 mouse retina^83^^–^^85^; once elicited, the RGCs displayed a regular multipeak firing pattern in clear contrast to that of the wild-type retina, which exhibited a single peak.^86^^,^^87^ This evidence lines up with observations from other immunocytochemistry studies on excised retinae.^67^^,^^88^ Using the same animal model, they showed a 50% reduction in the size of the dendritic field as well as in the total number of branching points of some of the RGCs analyzed that may explain said decrease in the sensibility to electrical stimulation in the indirect activation strategy. Similar results have been obtained from experiments in rd10, where RGCs exhibited higher activation thresholds to electrical stimulation compared to wild-type retinae.^89^

### The Relevance of the Survival of the RGCs

RGC death has also been reported to be a consequence of the retinal degeneration process, as documented in RGC population quantification experiments carried out in P23H-1 and Royal College of Surgeons (RCS) rats (another animal model of retinal degeneration).^62^ Similar findings were reported in rd1 mice, where this RGC loss was found in the peripheral region of the retina.^61^ However, there is extensive evidence in the scientific literature, from preclinical^90^^–^^92^ to clinical studies,^93^^–^^95^ that demonstrates that the retinal network is sufficiently preserved to enable functional visual restoration by electrical stimulation. Unavoidably, some of the RGCs perish during retinal degenerative disease progression. However, if the RGC survival rate is increased by any means, more neural targets will be available to be recruited by electrical stimuli, thus improving, presumably, the visual sensation perceived by implant recipients. Along these lines, neuroprotective strategies seek to preserve the neural tissue to avoid the harm caused by neurodegenerative diseases.^96^ Many different approaches have been demonstrated to have said neuroprotective effects in the retina. For instance, growth and neurotrophic factors have been used in clinical trials against retinitis pigmentosa, macular degeneration, and other ophthalmic diseases (see Fudalej et al.^97^ for a review). Other approaches, such as electrical^98^^,^^99^ or thermal^100^ stimulation of the retina, or the administration of plant-derived polysaccharides,^101^ among others, are also promising means to delay RGC apoptosis, thus extending the time window available for an optimized restoration of the visual function.

There is a need for developing more reliable techniques able to assess the number of viable RGCs, as they can assist with determining whether the preservation of RGCs can substantially benefit the visual percepts elicited from a retinal implant. Current techniques employed in clinical practice, such as optical coherence tomography (OCT) or perimetry, can only provide an estimation based on parameters that are indirectly related to the number of RGCs, leading to significant errors (see Smith et al.^102^ for a review). For example, although OCT imaging has been widely used, it lacks enough resolution to differentiate between RGC layers and other retinal layers, hindering the estimation of the real number of surviving RGCs. In the same way, perimetry is based on the patient's subjective perception of visual stimuli, a process that does not depend entirely on the RGCs, as visual perception is a complex process that involves many factors. The use of transgenic animals,^103^^,^^104^ adeno-associated viral vectors,^105^ or retrograde tracers^106^^,^^107^ to specifically label RGCs are examples of some of the techniques currently being used in research to track the RGCs in vivo, which can provide a direct measure of the RGC counts. While these techniques are useful in the evaluation of neuroprotective approaches in experiments with animal models, they cannot be applied to humans in the clinic.

## Changes in the Brain Produced by Retinal Degeneration

### Alterations in VC Molecular Dynamics and Light-Driven Information Processing

As described in the previous section, retinal degeneration entails severe modifications of the neural circuits that alter the activation patterns of the different neuronal populations. These neurons are responsible for encoding inputs to the visual system, even if these inputs are artificially introduced by electrical stimulation. Therefore, altered neural signals delivered from a degenerated retina via the optic nerve may also modify the way the visual information is processed in the brain by inducing a subsequent neural rewiring in higher visual centers.^24^

On that basis, a recent study on rd10 mice described, for the first time to our knowledge, the unbalances that exist in the excitation/inhibition mechanisms within the VC and LGN.^108^ Immunoblotting was carried out in wild-type and dystrophic cortical tissues using antibodies targeting the vesicular transporters of GABA and glutamate, among other neurotransmitters, showing statistically significant differences between the two experimental groups. Furthermore, nerve growth factor and brain-derived neurotrophic factor, two neurotrophins expressed within the central nervous system, have been found to present altered expression levels along the visual pathway in dystrophic RCS rats.^109^ As these neurotransmitters and neurotrophins have been shown to play essential roles in modulating cortical synaptic plasticity^110^ and neuronal survival and growth,^111^^,^^112^ respectively, alterations in the neural architecture of visual processing centers are expected to be found. Also, these changes might have direct consequences on how the visual information that arrives from the retina is processed.

**Figure 1.**
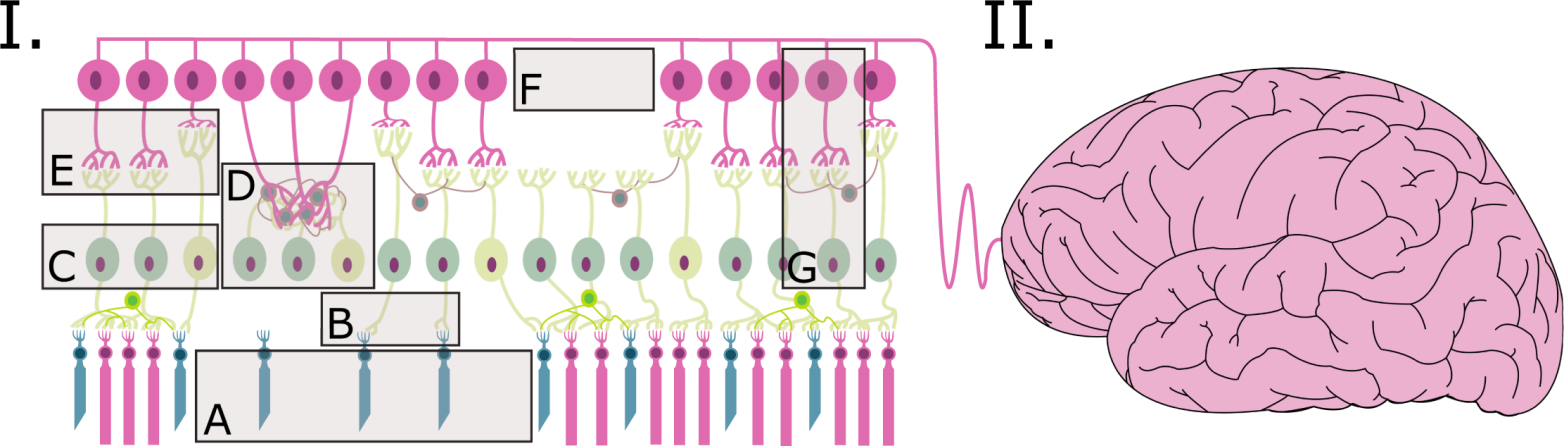
Schematic representation of the main alterations that occur during the course of retinitis pigmentosa that may have a direct implication in sight restoration via retinal prosthesis. (**I**) As the disease progresses, an important remodeling takes place in the retina that alters the neural circuitry. These modifications entail an important degradation of the processing and transmission functions of the retina, which eventually communicates abnormal information to the brain. (**A**) In the photoreceptor layer, rods die but cones can be still present. (**B**) Deafferented ON rod bipolar cells are now connected to the nearest cone available. (**C**) A general function shift occurs among the bipolar cells. ON-type bipolar cells become OFF-type, thus increasing the OFF-to-ON bipolar cells ratio. (**D**) Some cells that lose their synaptic connections tend to establish new connections with adjacent cells, thus forming neural tangles that deliver nonphysiologic information to RGCs and promote their activation. (**E**) RGCs modify their dendritic arbors, establishing new connections with bipolar cells. (**F**) A sectorial loss of RGCs causes a decrease in the amount of neural targets available for electrical stimulation. (**G**) RGCs display spontaneous activity as a consequence of an electrically coupled network established between AII amacrine and ON cone bipolar cells. (**II**) Retinal degeneration alters the entire visual pathway, including visual processing centers in the brain. Spontaneous activity of the visual cortex, quantitative alterations in neurotransmitters and neurotrophins affecting plastic dynamics, conversion of visual cortex neurons to process information from a different sensory modality, and cortical remapping are some of the changes described in the brain as a result of retinal degeneration.

Light-driven electrophysiologic VC responses originating in the degenerated retina have also been documented. For example, two in vivo studies were carried out in anesthetized rats using different visual patterns to evaluate light-driven responses in the VC. In the first one, smaller receptive fields and lower spatial and temporal tunings and orientation selectivity were reported^113^ in the VC of the S334ter-3, a retinal degeneration model caused by a rhodopsin mutation. In a second study, the processing of visual patterns, the latency of visual responses, and the amplitude of the responses to temporal changes in the luminance level were also reported to be altered in RCS rats’ VC.^114^ In addition, aberrant behaviors such as spontaneous hyperactivity has also been found in VC neurons of the S334ter-3 rat.^115^ Although the origin of this spontaneous activity remains unclear, it unavoidably resembles that observed in the RGCs of the degenerated retina.^70^^,^^116^ The similar behaviors observed in retinal and VC neurons might be explained in terms of nonphysiologic information generated in the diseased retina, transmitted by the surviving RGCs, and ultimately processed in the rewired VC, as represented in the example of Figure 1. This idea is also supported by a recent study wherein S334ter-3 rats had their vision restored via fetal retinal sheet transplant after complete vision loss. Note that in this case, dystrophic animals showed normal responses in primary neurons of the VC retinotopically matched to the transplanted region of the retina.^117^ This would suggest that the aberrant responses documented in VC neurons might originate from the transmission of the abnormal neural patterns in the degenerated retina, and therefore, the intrinsic electrophysiologic properties of VC neurons would be well preserved during the retinal degeneration process.

### Relevance of the Critical Period in Bionic Vision

Experience-dependent plasticity occurs throughout life. It is the basis of the learning process that takes place in the brain and allows for a progressive improvement and refinement in the performance of different tasks.^118^ Furthermore, during certain time windows in the development of different sensory modalities, the so-called critical period, a more prominent experience-dependent neural reconnection takes place. In humans, it occurs within the first years of life and can extend into adolescence.^119^^,^^120^ During these critical periods, neurons in the sensory-processing centers of the brain are more likely to establish new connections that can shape the neural circuitry upon the sensory inputs.^121^^,^^122^ However, once the critical period of a particular sensory function ends, the maturation process is considered completed, and the capacity that further experience has for inducing neuroplastic adaptive changes decreases dramatically.^123^^,^^124^

Contrast sensitivity, Vernier acuity (or the ability to discriminate spatial positional offset), sensitivity to global structure, and contour integration are some of the different visual properties that the VC can develop.^125^^,^^126^ Each of them, despite being processed in the same brain structure, has an independent sensitive period with unique duration and onset/offset times.^121^^,^^127^^–^^130^ The visual functions of the newborn are initially limited to the perception of basic visual features, but as the critical periods become available, experience-dependent visual functions progressively maturate using visual experience as a reference to establish new neural circuits.^124^^,^^131^ For this reason, missing or abnormally encoded visual sensations captured by a diseased retina during these sensitive visual development periods could drive the VC to specialize in the optimization of low-amplitude and noisy signals,^123^ hindering future visual restoration attempts. For example, there is a relevant case reported on a patient who lost sight after chemical and thermal corneal damage at the age of 3 and had vision restored after a corneal and limbal stem cell transplant 40 years later.^132^ It showed recovery of some basic visual features such as chromatic discrimination or object motion, but three-dimensional object perception and face recognition remained undeveloped even 10 years after surgery.^132^ A similar phenomenon has been reported in children with bilateral congenital cataracts, as they maintained visual deficits during their entire life even if the corrective surgery took place within their first month of life.^133^ In this regards, Beyeler et al.^38^ reviewed the relevance that the reawakening of the critical period, through pharmacologic means, might have in the neural adaptation process of the brain after sight restoration. In this work, the authors referred to the use of some drugs already approved that seemed to have enhanced neural adaptation in cochlear implants recipients. However, similar results have not been reported in bionic vision. They also highlighted not only the importance of modifying the neurochemical balances underlying neuroplastic mechanisms but also the essential role of epigenetics in the promotion of said cortical plasticity.^38^

It has to be noted that visual impairments caused by loss of photoreceptors are progressive and generally slow processes whose degeneration onset, pattern, and speed differ from person to person.^134^ For example, signs of early onset retinitis pigmentosa can appear during adolescence or adulthood.^134^ Even in those cases, the VC functions will have had enough time to complete a healthy development throughout a quasi-normal visual experience. For this reason, critical periods, in relation to the development of the VC, may not be a matter of concern regarding visual restoration approaches for the treatment of retinal degenerative diseases like retinitis pigmentosa, as relevant retinal remodeling typically occurs later.

### Retinal Electrostimulation and Adaptive Plasticity

Other plastic changes, yet with great impact in adaptation to the new sensory reality of the blind, still occur well beyond the end of the critical periods.^135^ For example, retinal receptive fields have been found to suffer cortical remapping due to retinal alterations such as those caused by lesions or degenerative diseases, as recently reported in a study by Ferreira et al.^136^ Using magnetic resonance imaging, Ferreira's team concluded that there was a topographic remapping of the peripheral visual field in the VC corresponding to the peripheral degeneration pattern of the retina. It appeared that the central retinotopic area of the VC enlarged and invaded peripheral regions, and this was more evident in patients with more severe conditions. Preservation of VC plasticity in adults with retinitis pigmentosa has also been assessed by measuring their ability to recover from monocular visual deprivation.^137^ Despite the long-term visual deprivation produced by the disease, VC plasticity caused by short-term changes in the visual inputs was still present, showing no differences with sighted individuals. Along these same lines, this idea has been also supported by a research work where the authors showed that the VC of the rd10 mouse is as susceptible to long-term potentiation as in wild-type individuals.^138^ These are just few examples of the enormous potential of the brain to cope with nonphysiologic neural information. Whether or not these types of neuroplastic mechanisms reported beyond the critical periods can assist the recipients of visual prostheses needs to be carefully considered.

Rehabilitation outcomes from patients implanted with the Argus II have shown that the training phase plays a critical role in the patient's interpretation of the visual perception elicited from a retinal prosthesis.^93^^–^^95^ Using functional magnetic resonance imaging (fMRI), increased blood oxygen level-dependent responses in the LGN and in the VC following a prolonged use of the Argus II retinal implant were found.^139^ Note that responses before implantation (or from subjects who did not spend long enough using the device) were significantly weaker. Furthermore, new training strategies involving labeling tasks of everyday objects significantly improved performance, both in blind patients implanted with Argus II^140^ and in sighted individuals under simulated prosthetic vision.^141^ Other retinal devices such as the Alpha IMS or the IRIS II have also achieved significant improvements in patients’ everyday tasks.^142^^,^^143^ However, despite the enormous progress over the past 20 years, bionic vision has not yet reached a level of success comparable to that of the cochlear implant.^144^ While there was great hope relating to the capacity of the brain to cope with artificially generated inputs to the visual system, scientific research has shown that such brain adaptation is not sufficient to obtain comparable results to those from the cochlear implant. Whether the learning process during rehabilitation directly modifies visual perception or just enhances the association between certain artificial stimulation patterns and previous memories of visual perception is still unknown. Thus, further studies analyzing brain activity in long-term retinal implant recipients are still needed to determine how the brain adapts to electrical excitation of the visual system from visual prostheses; this might be a key enabler for developing more effective rehabilitation strategies.

### Retinal Electrostimulation and Cross-Modal Plasticity

Lack of incoming input signals to sensory neurons, as in the case of blindness or deafness, triggers a rewiring process upon thalamocortical and corticocortical pathways where deafferented neurons are repurposed by the remaining sensory modalities (see Lee and Whitt^145^ for a review). In other words, the neuronal resources of the brain that were originally conceived to process a given sensory input can now be devoted to processing information arising from a different sensory modality. Far from causing some sort of unintelligible tangle of information within rewired processing centers, this neuronal reorganization leads to a better performance when dealing with tasks that originally involve absent or deprived senses. For example, subjects who have gone blind in early life tend to develop both auditory and tactile perception beyond the potential of sighted individuals: Braille readers have higher tactile spatial acuity compared to sighted persons,^34^^,^^146^ and in general, visually impaired subjects develop a better capacity to discern between different sound pitches.^147^^,^^148^ However, it is important noting that this improvement in the performance of tasks related to other sensory modalities is not only due to the recruitment of the deprived sensory area, as evidenced in fMRI^149^ and positron emission tomography^34^ studies, but also to the optimization of the previously existing neural pathways, a phenomenon known as compensatory plasticity.^145^ As an example of this, a mouse experiment demonstrated that thalamocortical inputs to auditory cortex (AC) were potentiated after visual deprivation, and inputs to the VC were potentiated after deafening, suggesting that thalamic synapses play an important role in the reinforcement of the pathways of spared sensory modalities.^150^

The human brain also presents inherent cross-modal connections between different sensory modalities. Certain thalamic and cortical structures, such as the pulvinars nuclei^151^ or the posterior parietal cortex,^152^ are known to be intrinsically multimodal. Similar cross-modal connections have been described and analyzed in other mammals. Along these lines, an experiment with rats showed that the lateral posterior nucleus, which would be the equivalent to the pulvinars nuclei in primates, projects onto the AC and becomes activated with signals coming from the superior colliculus, playing an important role in cross-modal modulation of auditory processing.^153^ These thalamic structures integrate information from different sensory modalities and project their connections onto the primary sensory cortices. In an experiment reported by Henschke et al.^154^ neuronal tracers were injected into the somatosensory, auditory, and visual cortices of the Mongolian gerbil. The study revealed that multisensory thalamocortical connections were abundant and exhibited a rapid development during the first postnatal month. Later, these connections were pruned away over brain maturation processes, and the pruning pattern was strongly influenced by early sensory experiences associated with critical periods. Furthermore, visually deprived individuals showed increased connections between thalamus and AC, as well as between AC and VC.^154^ Corticocortical connections between different sensory modalities are also inherently present in the brain, and they have been widely documented.^155^^,^^156^ Furthermore, there are some insights as to their functional implications. For example, in visually deprived rats, electrophysiologic recordings in the olfactory bulb registered stronger local field potentials compared to sighted individuals.^157^ Behavioral approaches developed in rats by a different study showed that visual deprivation impacted somatosensory modality as well,^158^ and somatosensory deprivation carried out in a another research work showed improved visual features in mice.^159^ The role of this intrinsic hyperconnectivity between different sensory centers and the capacity the brain has to potentiate these cross-modal connections has to be carefully investigated clinically in retinal prosthesis recipients, particularly in relation to the perceptual interpretation of the artificial inputs introduced into the visual system from an implant.

**Figure 2.**
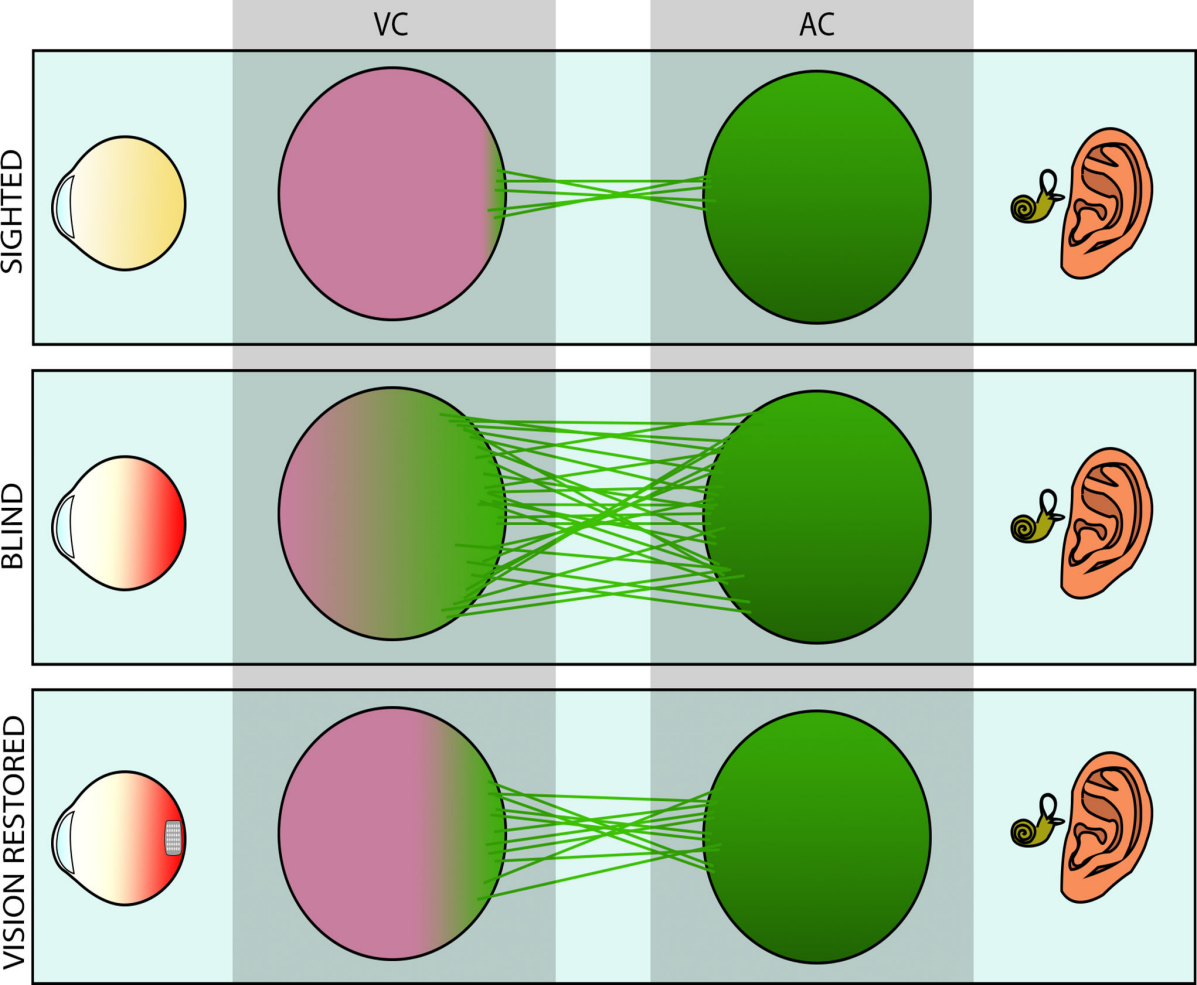
Schematic representation of the hypothetical neural rewiring that occurs in the brain during the progression of retinal degeneration. In normal vision (sighted), cross-modal projections of the neurons in the AC to the VC are scarce. Eventually, retinal degeneration progresses to a point that impedes normal vision (blind). Visual deprivation potentiates reliance on hearing, thus strengthening the cross-modal rewiring. Resumption of a visual input (vision restored) may retrieve some of the use of the VC to process artificially encoded visual information.

Whether or not the new configuration adopted by the sensory processing centers in the brain will assist with making better use of the delivered sensory information, in this case by bionic devices, remains unclear.^135^^,^^160^^,^^161^ Note, for instance, that it has been found, in deaf individuals, that there is an inverse correlation between the cross-modal activation level of the AC by visual inputs and the final performance of cochlear implants.^162^^,^^163^ These results suggest that the neural pathways of the VC expand over the AC in the absence of auditory input, taking over structures inherently devoted to sound processing. In the case of blindness, a similar situation wherein the VC gets partially ruled by the AC could happen too, as represented in Figure 2. However, in the case of blindness, some studies have reported the persistence of cross-modal connections between the visual and the AC^164^ after sight restoration.^165^^,^^166^ These cross-modal connections did not seem to alter the vision recovery process: the VC still retained the property to activate in response to auditory stimuli with similar strength, or even with stronger responses, than those reported during blindness.^166^ It is important to note that sensory perception in humans shows up as a collage of information coming from different sensory modalities that work together and share information with each other by exploiting previously referred inherent cross-modal connections.^167^ For instance, a recent experiment showed that Argus II implantees were able to use cross-modal mapping between auditory and visual location, thus increasing perceptual accuracy.^168^ This example suggests that cross-modal potentiation through training during rehabilitation may play a critical role in the improvement of the performance of retinal prostheses. Alternatively, cross-modal connections can be artificially manipulated to promote or impede said rewiring by using anodal or cathodal transcranial direct current stimulation (tDCS), respectively.^169^

## Mimicking the Physiologic Neural Code of the Retina

Electrical stimulation of remaining neurons through implanted devices has been demonstrated to be a successful solution to some untreatable diseases. This is the case for the sinoatrial node,^170^ spinal cord,^171^ gastrointestinal tract,^172^ or vagus nerve^173^ stimulation. All these treatments have undergone a years-long refinement process where the stimulus delivery was progressively adjusted to obtain the desired response. For example, first implanted pacemakers were output-only devices that had a fixed preprogrammed regular stimulation pattern, while the modern ones are highly sensitive devices able to read physiologic parameters as input, process the information, and elicit optimal closed-loop responses.^170^ Sensory prostheses such as cochlear^174^ and retinal implants^15^ have gone through, and are currently undergoing, the same input-dependent signal optimization process. But the ultimate goal in visual restoration research (i.e., restoration of normal sight) has encountered numerous limitations.^175^ Perhaps the most difficult challenge lays with sending, by electrically stimulating the retinal neurons, the correct neural information^28^ to higher visual centers in the brain, as the physiologic neural patterns to be mimicked resemble complex mosaics of highly encoded information.^40^ As previously mentioned, there are several types of retinal neurons involved in processing visual information. This information is eventually encoded and transmitted by the RGCs, located at the end of the neuronal cascade of the retina. There have been many attempts in trying to further understand the complex mechanisms governing neural encoding of vision.^176^^–^^179^ Among other types of research works, those aiming to characterize the RGCs have been instrumental in the development of visual prostheses. For example, a recent study aimed to characterize several human RGCs based on their transcriptomic profiles using retinal stem cells.^180^ Using noninvasive imaging techniques, other researchers sorted different RCG subtypes based on anatomic features.^181^ However, electrophysiologic approaches still rely on the use of animal models, with mouse^56^^,^^182^ and primate^183^ retinal neurons being the best morphologically and functionally characterized to date. Understanding of the RGC electrophysiologic properties has been the basis for the development of recent stimulation strategies that better replicate the neural patterns transmitted to the brain by retinal electrostimulation.

### In Vitro Studies

The amount of information that can be transmitted to the brain from a visual prosthesis is limited. Perhaps the quality of the visual percepts thus elicited can be substantially improved by delivering more physiologically realistic neural information to the brain.^28^ Given the hypothetical importance of replicating the neural code of the eye from retinal implants, the scientific community has also sought to devise new stimulation strategies able to preferentially activate the different neural pathways of the retina. The first attempts relied on attaining highly controllable single-spike responses from targeted RGCs. Fried et al.^184^ achieved this by delivering short-pulse duration stimuli (<0.15 ms) to an excised rabbit retina, thus avoiding signal reverberation arising from the neighboring neuronal circuitries involving bipolar cells. Furthermore, these short stimuli were demonstrated to work at rates below 250 Hz, enabling a method to closely control spike elicitation in retinal prostheses.^184^ Two years later, Sekirnjak and coworkers^185^ demonstrated the feasibility of achieving these single-spike responses in macaque-excised retinae using multielectrode arrays. The latency of the neural responses to electrical stimulations shown in this study were within the submillisecond range (around 0.19 ms), and subsequent responses could be elicited every 0.1 ms, thus demonstrating that high-precision temporal visual inputs could also be delivered to the primate's retina by these means. In addition, recent works by the same group have demonstrated the reliability of this stimulation paradigm to be used in retinal prostheses by mimicking the activation patterns of the retina elicited by a moving object.^49^ Furthermore, they have made relevant contributions to the understanding of how the activation of nearby electrodes may enable the elicitation of neural responses in the RGC population with high spatial resolution.^186^

While time-controlled spike elicitation provides accurate control over the spatiotemporal occurrence of neural spikes, classical biphasic stimuli in the absence of additional measures tend to recruit all neuron types indistinctly. In other words, these methods aimed at replicating the spatiotemporal properties of the neural patterns of retinal information. However, the preferential activation of the different information streams was not demonstrated until kilohertz-frequency current electrostimulation was applied.^31^^,^^48^ Ever since, many studies have focused on achieving independent electrical activation of the different RGC subtypes, to replicate the natural neural code driven by the RGCs in response to physiologic vision. For example, modulation of high-frequency electric current pulse trains (amplitude and frequency) has demonstrated a great potential to preferentially activate the ON and OFF pathways.^31^^,^^33^^,^^48^^,^^187^^–^^189^ In rabbit excised retinae, these ON and OFF RGC subtypes showed opposing activation patterns when applying high-frequency pulse trains with modulated amplitude.^31^ This study was further supported by a computational work^188^ and has been recently extended in an in vitro study using patch clamp on excised mouse retinae. In said research, a differential activation map was obtained after delivering a repertoire of stimuli with different current amplitudes and frequencies to four different RGCs subtypes: OFF-sustained, OFF-transient, ON-sustained, and ON-transient.^190^ Kameneva and coworkers^189^ found that ON cells were more likely to activate at higher frequencies than OFF cells, and similar results were also reported regarding sustained versus transient cells respectively. Furthermore, a specific range of current amplitudes and frequencies was defined for three out of the four cell types analyzed in the experiment (OFF-sustained, OFF-transient, and ON-transient), suggesting that they can be differentially activated.^190^ In general, OFF RGCs appeared less sensitive to changes in the stimulation parameters than ON RGCs.^191^ Along these lines, RGCs have also shown differential responses to low- and high-frequency sinusoidal electric stimulation, which can be added to the set of stimulation tools available for the replication of the neural code of the retina.^192^ Variations in the pulse duration have also been found to allow for selectively activating certain neuron types.^191^^,^^193^ Perhaps, the specific cell morphology and the electrophysiologic properties of the different RGC types can explain the underlying mechanisms that allow preferential activation of the different pathways.^194^^–^^196^ Although these studies reveal great significance for the field, the relatively high current amplitudes required in some of these experiments have also shown to be hardly applicable in current retinal implants, as they can bring the neurons toward their inhibitory threshold,^197^^,^^198^ potentially deterring the formation of visual percepts.^140^

Indirect activation of the RGCs through stimulation of bipolar cells has also been proposed as an alternative method to elicit visual perception via visual prosthesis. As opposed to direct activation, indirect activation has the potential to produce more natural RGC responses since they are being elicited by the neurotransmitters delivered by bipolar cells as it takes place in physiologic vision.^199^ However, Im and Fried^199^ showed that, despite the responses generated in ON RGCs by indirect activation correlates to those obtained using visual stimuli, OFF RGCs showed poor correlation. Furthermore, a recent study explored the variability of the RGCs network-mediated responses in the rd10 mouse with age, demonstrating that the consistency of the responses decreased along with the grade of retinal degeneration.^200^ The benefits and drawbacks of direct and indirect activation of the RGCs are still a matter of debate,^201^ and further research is needed to clarify which option suits better each particular clinical case.

### In Silico Studies

Computational studies have been broadly used as an alternative tool to virtually explore the effects of altering the stimulation parameters in bionic vision. In these approaches, morphologic and biophysical neuronal data are typically collected from in vitro studies and are normally used to refine the model parameters and to validate research outcomes. In particular, these techniques have been utilized to explore the stimulation parameters able to provide optimal RGC selectivity, typically by modifying the amplitude and frequency of high-frequency electric current pulses. Results from the first studies indicate that preferential activation of the ON and the OFF pathways arises from the differences that exist in the morphology of the soma, the dendritic tree, and the neural axon, as well as from the different properties of the ionic channels of each RGC type.^183^^,^^188^ Further computational studies combined a closed-loop algorithm able to progressively modulate the electrical current in order to obtain optimal parameters that maximize selective recruitment of ON and OFF cells.^33^ In another in silico study, Guo and coworkers^32^ presented a novel approach involving two stimulation targets for mimicking physiologic responses: a first group of electrodes would activate both ON and OFF RGCs simultaneously, while the second group, placed near the optic nerve, would alter the traveling neural signals previously elicited. APs arriving from a certain RGC type would be selectively suppressed by the second stimulus, thus allowing for the ultimate transmission to the cortex of the signals arising from one type of RGC only. Note that the ON and OFF RGCs are not the only neuronal types analyzed in the in silico studies found in the scientific literature. Other RGC types and retinal neurons have also been modeled and characterized, including parasol and midget RGCs,^202^ D1-bistratified and A2-monostratified RGCs,^203^ or bipolar cells.^203^^–^^205^ By exploiting the different characteristics of the various RGC types, these studies allow for exploring new stimulation strategies that can potentially improve retinal prosthesis outcomes. However, to the best of our knowledge, none of the studies using high-frequency stimulation have taken the leap to confirm the effectiveness of these strategies in vivo, as summarized in the Table.

**Table.** Principal Studies on the Replication of the Neural Code of the Retina Using Electrical Stimulation

Research Study	In Vitro	In Silico	In Vivo
Fried et al. (2006)^184^	X		
Sekirnjak et al. (2008)^185^	X		
Cai et al. (2013)^48^	X		
Twyford et al. (2014)^31^	X		
Jepson et al. (2014)^49^	X		
Jepson et al. (2014)^186^	X		
Twyford et al. (2014)^31^	X		
T. Guo et al. (2014)^188^		X	
Werginz et al. (2015)^205^		X	
Weitz et al. (2015)^241^	X		
Kameneva et al. (2016)^189^	X	X	
Hosseinzadeh et al. (2017)^242^	X		
Guo et al. (2018)^33^	X	X	
Kotsakidis et al. (2018)^187^	X	X	
Im et al. (2018)^193^			
Haq et al. (2018)^243^	X		
Guo et al. (2018)^206^		X	
Tong et al. (2019)^244^	X		
Tong et al. (2019)^245^	X		
Ryu et al. (2019)^210^			X
Muralidharan et al. (2020)^190^	X		
Werginz et al. (2020)^208^		X	
Song et al. (2021)^202^		X	
Paknahad et al. (2021)^203^		X	
Paknahad et al. (2021)^204^		X	
Paknahad et al. (2021)^203^		X	

To date, new strategies to preferentially activate different types of RGCs have been studied in vitro and/or in silico mainly.

Computational models have been used not only to characterize the response of diverse RGC types to different stimulation strategies but also to unravel the biological basis of certain electrophysiologic phenomena related to neuronal electrical stimulation. For example, a recent study provided some insights on the stimulus strength–dependent suppression inherently linked to high-frequency stimulation.^206^ The authors suggested that the local hyperpolarization that occurs near the stimulation electrode is produced by outward sodium currents and that this process was directly involved in the mechanisms underlying this phenomenon.^206^ Furthermore, the AP activation threshold of α-RGCs, another RGC type found in mammals,^207^ has also been characterized in mice.^208^ They found that the axon initial segment was the region with the lowest activation threshold and that the value of this threshold varied along its length and the relative concentration of sodium channel present in the region. In contrast, neuron properties such as the size of the cell or the dendritic tree showed minor influences regarding the activation threshold.^208^

Along the same lines as in the in vitro research, the properties of indirect activation of the RGCs have also been studied in silico. Using computational modeling frameworks based on real physiologic data, RGC network-mediated responses have been simulated to explore its potential as an alternative stimulation tool to be used in visual prosthesis. In an attempt to better understand the electrophysiologic mechanisms governing indirect activation of the RGCs, Paknahad et al.^204^ have analyzed the effects that different stimulation parameters have on the activation threshold of the bipolar cells. They have also studied the role that synaptic membrane channels can play in the subsequent activation of the RGCs.

### In Vivo Studies

There is also a body of literature on retinal electrostimulation research conducted in experimental animals to analyze the neural patterns elicited in higher visual centers. Many of these studies aimed at shedding light on whether these artificially elicited neural patterns can mimic those that appear in healthy vision; substantially different neural patterns may not be correctly interpreted by VC neurons and can potentially trigger subsequent neuroplastic changes. Initial studies performed in feline models aimed at investigating the extent of the cortical activation that follows the delivery of electrical stimuli from different prototypes of retinal implants,^209^^–^^212^ focusing on the spatial dimension of the neural code of the retina. Along these lines, subsequent studies targeted a repertoire of current steering techniques to improve activation of the neural targets by modifying the return configuration^140^^,^^213^^–^^216^ or by overlapping the electric fields produced by concurrent stimuli.^140^^,^^197^^,^^216^^–^^218^ Similar approaches have been also addressed in murine models.^219^^–^^221^ However, there are limited studies addressing the preferential activation challenge required for the replication of the neural code of the retina. A recent study by Ryu et al.^210^ has reported out-of-phase ON/OFF cortical responses in mice following low-frequency transcorneal retinal electrostimulation. However, as previously stated, many of the findings on the preferential activation of the different types of RGCs using high-frequency electrical stimulation remain to be verified in vivo.

## Discussion

A number of therapies aiming to restore sight have emerged in the recent years. From electrical stimulation to the edition of the defective genes that cause blindness, these strategies share a common goal: the delivery of high-quality vision. Perhaps, some of the difficulties these approaches are facing can be explained in terms of remodeling the surviving neurons suffer during the course of the disease^60^^,^^222^ and their subsequent effects downstream. Some studies reported significant alterations of the neural pathways, both morphologic^67^^,^^88^ and electrophysiologic.^86^ However, other studies showed that the intrinsic properties of individual RGCs are preserved during the disease.^82^^,^^223^ A similar controversy exists in the scientific literature regarding the survival of the RGCs: while some experiments showed that there is a notable loss of RGCs in dystrophic animals,^61^^,^^62^ other studies found no significant differences with the sighted controls.^91^^,^^224^ Note that there are important dissimilarities in the experimental approaches of these studies that may lead to conflicting conclusions such as the mutations causing the retinal decay, the severity of the disease, the age at which symptoms began, the genetic background of the animals used in the experiments, the RGC sorting criteria, or the recording and stimulation techniques, among many others, and therefore, they need to be carefully interpreted. Along these same lines, the family of retinal degenerative diseases is broad, and each individual experiences a different progression of visual impairment, even in the presence of the same genetic mutations.^134^ Hence, the sought-after improvement in the quality of vision may come along with more personalized rehabilitation strategies that allow for exploiting the neural resources remaining in the rewired retina. However, available tools to measure the degree of preservation of said neural resources are limited in the clinic and can only provide an indirect assessment. Perhaps, the combination of a robust arsenal of electrical stimulation strategies and use of artificial intelligence methods to analyze cortical electroencephalographic information^225^ can assist with better tuning the next generation of bionic eyes.

Death of photoreceptors caused by degenerative diseases alters the neural behaviors of the remaining neurons in the visual system: multispike APs, higher electrical activation thresholds, function change of the different information streams, and so on. It is worth highlighting that a relevant portion of the RGCs^116^ and the neurons of the VC^115^ exhibit spontaneous activity in dystrophic individuals. Note that if the RGCs are not rescued in a timely fashion, they may form new anomalous retinal circuitries, the so-called micro-neuromas.^60^^,^^73^^,^^226^ These are complex structures found in the degenerated retina that self-fire even in the absence of an input from the photoreceptors, as mentioned previously. The neural signals from these circuitries lack visual information and can eventually reach the VC, causing the meaningless visual percepts documented in testimonies from blind people.^74^ An early intervention that preserves said increased neuronal rewiring capabilities of the retinal neurons while impeding the formation of aberrant circuits may benefit the ultimate outcome of retinal prostheses. While slowing down the progression of degenerative diseases is not always feasible, perhaps the reintroduction of inputs into the visual system might help, to some extent, with restoring the original circuitry. Nevertheless, visual restoration by means of retinal implants takes place only when a substantial degree of visual perception is lost. Therefore, these abnormal self-firing patterns are likely to occur in the majority of the implant recipients, and consequently, visual prostheses need to deal with this phenomenon. The development of complementary electrical stimulation strategies, such as the use of subthreshold prestimuli^78^ or high-frequency electric current, may assist with abolishing said abnormal activity, thus improving the stimulation efficiency. Along the same lines, it has to be noted that some retinal prostheses are currently targeting only a small portion of the VC, and therefore, preservation of the overall RCG count might not be excessively relevant in the sought for high-quality vision. In addition, it is worth mentioning that despite the RGC count decay that occurs normally in the nonpathologic aging retina, visual sensations are not substantially altered. Although RGC deaths in both cases, the aged and the diseased retina, are not equivalent, the electrical activation of a viable subpopulation of the RGCs is expected to be sufficient for providing good vision.

Despite the relevant neural remodeling that takes place in the diseased retina, retinal prostheses have demonstrated that the remaining RGCs can deliver artificially encoded neural information to the brain, which forms rudimentary visual percepts.^143^ Note that some stimulus waveforms can activate not only the RCGs but also other cells in the retinal network. Together, the indirect activation of a rewired retinal network, the spontaneous activity caused by the aberrant neural circuitries, and the unselective activation of the different RGC types can explain the relatively low-quality percepts induced from current retinal implant technologies. Although the real impact on the quality of the visual percepts thus elicited has not been fully assessed yet, it is highly plausible that these nonphysiologic neural patterns are not fully understood by the higher visual centers that produce visual percepts. The effective adaptation to nonphysiologic neural patterns reported in cochlear implant recipients seems to not follow the same success road in bionic vision. Therefore, new stimulation strategies adapted to a retina that is no longer able to process the information in a physiologic way should be investigated to allow the delivery of more realistic neural messages.

The VC can reorganize its retinotopic mapping during visual impairment, arguably to compensate for visual deficits.^136^^,^^227^^,^^228^ This reorganization may be due to loss of normal retinal signaling arriving at the VC and causing subsequent synaptic rewiring. Nevertheless, other studies on patients with macular degeneration and retinal lesion experiments on macaques declare not having found any evidence of said retinotopic remapping.^229^^–^^231^ Taking into account the high neuroplastic capacity of the VC, the potential it might have during the reintroduction of the visual input merits thoughtful considerations. Thus, further complementary efforts may be focused on the understanding, manipulation, and perhaps promotion of these biological mechanisms to improve the ability of the brain to cope with artificial neural information, for example, by using cognitive enhancement strategies such as the delivery of transcranial electric current.^169^^,^^232^ At present, current noninvasive technologies can provide only limited information on the role neural plasticity plays in the adaptation to a retinal prosthesis. Note that most research results on brain plasticity arise from animal models, both in vivo and in vitro. Although neural plasticity remains a hot topic in neuroscience, there are important gaps in the study of the rewiring processes that follow the reintroduction of inputs into the visual system. Therefore, there is an important need for further translational studies able to shed light on the mechanisms underlying these reconnections. The development of functional models is of particular interest as they can shed light on the correlation between electrophysiologic observations and the ultimate visual perception, bringing basic research closer to the bedside.

The cross-modal plastic changes that occur following visual impairment appear as a natural adaptation mechanism to the lack of vision.^34^^,^^146^^,^^148^ Brain areas that go “unused,” in this case by the lack of visual input, are devoted to processing other sensory modalities. This was evidenced by the activation of visual areas by tactile^34^ or auditory stimulation.^149^ Both thalamocortical and corticocortical pathways are known to be involved in this process.^150^^,^^154^ However, it is not clear whether these types of plastic changes hamper or benefit visual rehabilitation. On the one hand, the ability of the visually impaired to understand other sensory modalities increases notably; many get accustomed to a new sensory world, and some may even become reluctant to rely on the phosphenized vision from bionic devices. On the other hand, an increased connectivity of the visual system may facilitate further remodeling after visual restoration. Some cochlear implant research studies suggest that activation of AC by visual inputs translates into an unfavorable adaptation to the implant.^162^^,^^163^ In visual prosthesis, the scarce evidence available at present seems to suggest that the opposite may be true.^166^ Many concurrent physiologic phenomena remain to be further analyzed, particularly regarding the plastic changes that the visual system experiences during the natural history of the disease. It appears to be an important research challenge to isolate and determine the impact each of these physiologic processes has in visual rehabilitation. In particular, the role of cross-modal plasticity needs to be unscrambled. Perhaps then, new approaches to either promote or inhibit this cross-modal rewiring may be devised to optimize the interpretation of the poorly encoded visual information delivered from retinal implants.

Down the same road, the delivery of more realistic neural messages is expected to improve the visual percepts elicited from visual prostheses, as the brain will be able to make better interpretations. Although this hypothesis has not been confirmed yet, many researchers in the field work toward eliciting more physiologically relevant neural responses. Unfortunately, our ability to replicate the neural code of the eye is still limited. The different approaches investigated to date range from accurately timing the elicitation of single APs from the RGCs^184^ to the delivery of modulated high-frequency pulse trains to preferentially activate different RGC types.^31^^,^^33^^,^^48^^,^^187^^–^^190^^,^^197^ An arsenal of different stimulation tools is being developed by different research groups worldwide. However, the visual code of the retina is complex, our knowledge is limited, and, more important, the diseased retina is rewired dynamically, and some neurons may change their function over time. In addition, most of these studies have been conducted either in vitro using excised retinae or in silico using computational models of certain retinal neurons only, whose parameters were determined from healthy retinae.^33^^,^^188^^,^^203^ There is therefore an urgent need to determine whether these stimulation strategies, particularly the use of high-frequency stimulation, have similar outcomes in vivo, as this is the previous step to determine whether these research efforts will translate into better visual perception. In doing so, it is important to verify if said preferential activation replicates in models of the disease. Note, for example, that the identification of the ON and OFF RGCs is based on their response to visual stimulation; the question on whether preferential activation of these pathways occurs in the fully degenerated retina encounters important limitations. It is also worth noting that most (if not all) bionic vision stimulators to date stimulate at a relatively slow rate and rely on the delivery of constant-current pulses, preventing the clinical evaluation of some strategies that might require the use of custom current waveforms. This has been from necessity since the visual scene cannot be refreshed quickly enough owing to limitations of the stimulator, because frequencies around a few Hertz do not produce physiologic outcomes consistent with useful vision. If either/both of these continue into the future, the “neural message” referred to here may not be possible to deliver.

In modern cochlear implants, the explanation for why they work extraordinarily well is still unclear. Thousands of cilia are replaced by 22 relatively course metallic electrodes that deliver constant-current biphasic pulses to the auditory system.^14^ The brain then learns to construe the neural messages thus delivered to produce a functional perception.^233^ In congenitally deaf children, an early implantation, particularly in the prelingual phase,^234^ has been demonstrated to aid with the development of speech and language skills.^235^ However, if implanted beyond approximately 7 years of age, the performance of the cochlear implant seems to decay as the critical periods end. The brain seems either to make sense out of artificially encoded neural messages in postlinguistically deaf adults or to form neural circuitries to process said neural messages in prelinguistically deaf implantees. Unfortunately, similar phenomena have not been observed in retinal implant recipients. Why one prosthesis works so well and the other is still facing important limitations remains a critical question in the field that cannot be answered based on previous studies. While there are some kinds of deafness that appear at birth and can be successfully treated with a cochlear implant, the types of visual impairment that can benefit from retinal implant are typically caused by degenerative diseases. Most people with retinal degeneration preserve sufficient vision until adulthood. Hence, candidacy for such an implant can only be considered after the critical periods of the visual system have closed. Therefore, progress in the understanding of the plastic mechanisms that could occur if a retinal prosthesis was implanted before closure of said critical periods can only come from studies in functional models of the disease, as referred to previously. However, it has to be noted that part of the success of the cochlear implant can be explained in terms of the relatively simple neural signals required for the interpretation of speech; the appreciation of music remains a comparable challenge to those in visual prosthesis.

Visual prosthesis has the potential to restore vision, particularly for those cases caused by retinal degenerative diseases. These diseases produce a cascade of neural rewiring along the visual system. To date, the implication of these neuroplastic changes in the restoration of sight is poorly addressed in the scientific literature. In addition, the neural messages—that is, the neural patterns that travel downstream the RGCs—delivered from retinal implants differ substantially from those in physiologic vision despite the important efforts in the design of new stimulation paradigms able to preferentially activate the different retinal neural pathways. In other words, electrical stimuli delivered at the remodeled retina elicit abnormal neural messages that get subsequently processed by a brain that has been reconnected, among others, to better process other sensory inputs. Unfortunately, two important questions remain to be answered: (1) whether altering neural plasticity, by promoting or inhibiting neural rewiring, can benefit visual restoration and (2) if more physiologically relevant neural messages, but still different from health vision, can elicit more meaningful visual percepts. Murine models have been used extensively to investigate similar neuroplastic phenomena and therefore seem to be an excellent resource to answer these questions. A repertoire of genetic, chemical, and molecular tools that can target specific pathways or neural structures are available. In addition, it is possible to conduct electrophysiologic recordings in both anaesthetized^50^ and behaving animals.^237^^,^^238^ These animals can be trained following any of the learning paradigms (classical or instrumental conditioning) to develop conditioned responses to visual perception that can be measured electrophysiologically as an indirect indicator of visual perception. The first question requires strategies to alter the neuroplastic properties of the visual system, such as the delivery of tDCS^169^ or the actuation over the expression of the Otx2 gene, among others, as this is known to play an important role in visual plasticity.^238^^,^^239^ Before the second question can be tackled, there is a need for demonstrating that the neural responses observed in the excised retina produce more physiologically relevant responses in the VC. Note that the proximity of the electrode to the target neuron seems to be a relevant parameter in the performance of preferential activation of RGC subtypes,^32^^,^^33^^,^^188^^,^^206^^,^^240^ and therefore, these observations may not be consistently replicable in vivo. In addition, the use of artificial intelligence in closed-loop experiments may assist with finding stimulation parameters able to elicit more physiologic patterns. The ultimate answer to these questions may arise from the clinical evaluation of the next generation of retinal prostheses, should these devices have the capacity to deliver custom current waveforms.
